# Facilitators Associated With Building and Sustaining Therapeutic Alliance in Advanced Pediatric Cancer

**DOI:** 10.1001/jamanetworkopen.2021.20925

**Published:** 2021-08-20

**Authors:** Erica C. Kaye, Sarah Rockwell, Cameka Woods, Monica E. Lemmon, Karen Andes, Justin N. Baker, Jennifer W. Mack

**Affiliations:** 1 Division of Quality of Life and Palliative Care, Department of Oncology, St Jude Children’s Research Hospital, Memphis, Tennessee; 2 Rollins School of Public Health, Emory University, Atlanta, Georgia; 3 Department of Pediatrics, Duke University, Durham, North Carolina; 4 Dana-Farber Cancer Institute, Boston, Massachusetts; 5 Department of Pediatrics, Boston Children’s Hospital, Boston, Massachusetts

## Abstract

**Importance:**

Therapeutic alliance is a core component of patient- and family-centered care, particularly in the setting of advancing cancer. Communication approaches used by pediatric oncologists to foster therapeutic alliance with children with cancer and their families are not well understood.

**Objectives:**

To identify key oncologist-driven facilitators associated with building and sustaining therapeutic alliance in the setting of advancing pediatric cancer and to develop a framework to guide clinical practice and future investigation of therapeutic alliance.

**Design, Setting, and Participants:**

In this qualitative study, serial disease reevaluation discussions that occurred in the clinic, inpatient hospital, or off campus via telephone were recorded among pediatric oncologists, patients with high-risk cancer, and their families across 24 months or until death, whichever occurred first, from 2016 to 2020. This analysis focused on recorded discussions for pediatric patients who experienced progressive disease during the study period. Content analysis was conducted across recorded dialogue to derive inductive codes and identify themes. Participants were patient-parent dyads for whom a primary oncologist projected the patient’s survival to be 50% or less, all family members and friends who attended any of their recorded disease reevaluation conversations, and their oncologists and other clinicians who attended the recorded discussions.

**Results:**

A total of 33 patient-parent dyads were enrolled and followed longitudinally. From this cohort, 17 patients experienced disease progression during the study period, most of whom were female (11 [64.7%]) and White (15 [88.2%]) individuals. For these patients, 141 disease reevaluation discussions were audio recorded, comprising 2400 minutes of medical dialogue. Most children (14 [82.4%]) died during the study period. A median of 7 disease reevaluation discussions per patient (range, 1-19) were recorded. Content analysis yielded 28 unique concepts associated with therapeutic alliance fostered by oncologist communication. Ultimately, 7 core themes emerged to support a framework for clinician approaches associated with optimizing therapeutic alliance: human connection, empathy, presence, partnering, inclusivity, humor, and honesty.

**Conclusions and Relevance:**

In this qualitative study, pediatric oncologists used diverse communication approaches associated with building and deepening connections across advancing illness. These findings offer a framework to support clinical and research strategies for strengthening therapeutic alliance among pediatric oncologists, patients, and families.

## Key Points

**Question:**

What communication strategies do oncologists use that are associated with building and sustaining therapeutic alliance in the context of advancing pediatric cancer?

**Findings:**

In this qualitative study, analysis of 141 disease reevaluation discussions representing 17 patient-parent dyads revealed 28 unique approaches used by oncologists that were associated with fostering therapeutic alliance. Ultimately, 7 themes emerged as strategies associated with optimizing therapeutic alliance: human connection, empathy, presence, partnering, inclusivity, humor, and honesty.

**Meaning:**

In this study, pediatric oncologists used diverse communication approaches to deepen connections across advancing illness, with 7 core themes supporting a framework for future clinical and research work to strengthen therapeutic alliance among oncologists, patients, and families.

## Teaser

This qualitative study identifies key communication strategies used by oncologists to build and sustain therapeutic alliance with children with advancing cancer and their families, developing a framework to inform clinical practice and future investigation of therapeutic alliance.

## Introduction

Patients with cancer and their families experience profound physical, psychological, and existential suffering across the illness trajectory.^1,2,3,4^ Development of therapeutic alliance between the patient or family and the oncologist may help ease the pain.^5,6,7^ Therapeutic alliance refers to the nature and strength of an affective bond between the patient or family and the clinician in collaboration toward shared goals.^8,9,10,11^ Among adult patients with advanced cancer, a strong therapeutic alliance with the oncologist has been associated with emotional acceptance of terminal illness and decreased invasive interventions at end of life.^5^ For young adults with cancer, the patient-oncologist alliance also has been shown to be protective against suicidal ideation.^12^ In addition, a positive patient-oncologist therapeutic alliance appears to benefit caregivers of adult patients with cancer across the illness course and during bereavement; more specifically, a patient’s report of a strong therapeutic alliance has been shown to be associated with a caregiver’s report of better social function, health-related quality of life, and emotional well-being following the patient’s death.^6^

As proposed by the National Cancer Institute^13^ and further validated in pediatric cancer,^14^ relationship building is considered one of the core functions of patient-centered communication.^15^ Most parents of children with cancer form trusting relationships with their child’s oncologist, and development of trust is associated with parent perception of high-quality physician communication.^16^ Fostering therapeutic alliance through patient-centered communication is particularly critical in the context of cancer relapse or progression, when children and families navigate stressful decisions.^17^ Unfortunately, poor outcomes have been shown to threaten therapeutic alliance, with parents reporting decreased trust in oncologists in the setting of advancing disease.^16^

Although high-quality oncologist communication is known to be important for therapeutic alliance,^16^ specific communication approaches used by oncologists to facilitate therapeutic alliance in pediatric cancer are not well understood. The U-CHAT (Understanding Communication in Healthcare to Achieve Trust) trial was designed to better understand the evolution and impact of communication across the advancing pediatric cancer trajectory.^18^ In this prospective, longitudinal study, serial disease reevaluation conversations among pediatric oncologists, patients with high-risk cancer, and their families were audio recorded across the illness course to the time of death. The primary aim of this study was to characterize the evolution of communication strategies used by pediatric oncologists across advancing illness. In this article, we present the findings from a secondary aim of that study: to identify and describe oncologist approaches for promoting therapeutic alliance, with the goal of developing a framework to organize emerging constructs and better understand potential facilitators for alliance to target in future research.

## Methods

The protocol for this qualitative study was developed by an interdisciplinary team of pediatric oncology and hospice and palliative medicine experts in collaboration with a bereaved parent steering council; it was reviewed and approved by the institutional review board at St Jude Children’s Research Hospital in Memphis, Tennessee. Data were collected between 2016 and 2020. We present study methods and findings following the Consolidated Criteria for Reporting Qualitative Research (COREQ) reporting guideline and checklist.^19^ All participants provided written or verbal informed consent that was obtained in a manner consistent with the Common Rule requirements. No one received compensation or was offered any incentive for participating in this study.

Details about the study protocol and feasibility and acceptability metrics for enrollment and capture of longitudinal data have been previously published.^18,20^ In brief, we enrolled a convenience sample of 6 pediatric oncologists who provided care to patients with solid tumors at an academic pediatric cancer center and obtained verbal informed consent. We then enrolled 4 to 6 patient-parent dyads with poor prognoses per oncologist. Eligible patients were aged 0 to 30 years, diagnosed as having non–central nervous system solid tumors, and considered to have poor prognosis, with the primary oncologist estimating survival of 50% or lower. Eligible parents or guardians were legal caregivers aged 18 years or older with English language proficiency who planned to be present for disease reevaluation discussions. Eligible patient-parent dyads were identified by the research team through review of outpatient clinic schedules and institutional trial lists, and permission to approach dyads was requested from the primary oncologist. A research team member obtained written informed consent from patients and caregivers during a clinic visit that did not include a disease reevaluation discussion; patients aged 12 years or older provided formal assent, and patients aged 18 years or older and parents provided consent. Demographic and disease-related information were extracted from the electronic medical record.

Patient-parent dyads were followed prospectively, with serial disease reevaluation discussions among patients, their family members and friends, and oncologists and other clinicians recorded in the clinic, hospital, or off campus via telephone across the illness course until death or 24 months from disease progression on study, whichever occurred first. The present work focuses on analysis of recorded discussions for dyads who experienced progressive disease during the study period.

To characterize key components of therapeutic alliance building in advancing pediatric cancer, we performed content analysis of recorded medical dialogue. We wanted to approach the data with openness to allow the dialogue to inform the findings; however, we also started with a priori constraints that shaped the lens through which we assessed the data (ie, we wanted to focus on clinician behaviors that served as potential facilitators of therapeutic alliance), precluding use of a traditional grounded theory approach. A research team representing medical and nursing perspectives across pediatric oncology and palliative medicine (Table 1) reviewed the literature related to therapeutic alliance in oncology; finding little consensus for fundamentals of alliance building in pediatric cancer care, we used an inductive approach^21^ to identify oncologist communication approaches to foster affective bonds with patients and families. Two researchers (E.C.K. and S.R.) repetitively listened to audio recordings, conducted extensive memo writing, and used raw data to inform development of codes, code definitions, and salient examples related to therapeutic alliance building.^22^ Additional researchers (C.W. and J.N.B.) reviewed recorded content and provided feedback in iterative cycles of codebook development. As facilitators were identified, we reviewed existing frameworks when possible to help codify concepts. For example, for expressions of empathy, we referenced the naming, understanding, respecting, supporting, and exploring framework for navigating emotions.^23,24^ However, through the processes of memo writing and inductive code development, we created definitions for each code based on the concepts emerging from the dialogue. The codebook was finalized following deep review of sufficient raw data to reach saturation, with no new therapeutic alliance concepts emerging from the recorded dialogue. The codebook is presented in Table 2.

**Table 1.** Research Team Attributes and Qualifications

Author	Attributes and qualifications
E.C.K.	Female physician-scientist with a medical degree, a master’s degree in public health, graduate-level training in qualitative research methods with a focus on communication science, and clinical training and practice in pediatric hematology-oncology and hospice and palliative medicine
S.R.	Female nurse-scientist with a master’s degree in public health, graduate-level training in qualitative research methods, and clinical training and practice as a pediatric oncology nurse and an advanced practice provider
C.W.	Female research associate with formal MAXQDA training and expertise in qualitative research methods
M.E.L.	Female physician-scientist with a medical degree, graduate-level training in qualitative research methods with a focus on communication science, and clinical training and practice in pediatric neurology and neonatal neurology
K.A.	Female scientist with a PhD degree in qualitative research methods and extensive experience with teaching and conducting qualitative research
J.N.B.	Male physician-scientist with a medical degree, extensive clinical and research expertise related to difficult communication in oncology, and clinical training and practice in pediatric hematology-oncology and hospice and palliative medicine
J.W.M.	Female physician-scientist with a medical degree, a master’s degree in public health, extensive research expertise in communication science, clinical training in pediatric hematology-oncology and hospice and palliative medicine, and practice in pediatric hematology-oncology

**Table 2.** Codes and Definitions Derived From Raw Dialogue Data

Code	Definition
**Human connection**	
Remembering	Oncologist recalls information, unprompted, that is personal or important to the patient’s or family’s life
Sharing	Oncologist contributes personal information about themselves or their life in an effort to find common ground with a patient or family, such as character, emotions, personal life, or work habits
Friendly conversation	Oncologist use of small talk that does not include symptom discussion, treatment plan, medical care, emotional support, etc; includes back-and-forth small talk between parent and oncologist
Affection	Any time the oncologist, parent, or patient expresses a sentiment or feeling of fondness toward each other
**Empathy**	
Standing in another’s shoes	Oncologist uses empathetic statements to respond to emotions; includes validation of emotions and sharing grief; may also include countertransference (ie, ability to imagine oneself in the patient’s or family’s position)
Naming	Naming the emotions displayed by the patient or family
Understanding	Acknowledging and appreciating the patient’s or family’s situation; validating emotions
Respecting	Offering praise whenever appropriate; oncologist provides statement of reassurance and encouragement to parent or patient
Supporting	Expressing concern and a willingness to help
Exploring	Giving the patient or family an opportunity to talk about whatever they are feeling or processing; exploring sources of conflict (eg, guilt, grief, culture, family, and trust in medical team); exploring values behind decisions; includes a probing question
Saying sorry	Oncologist uses the phrase “I am sorry” or synonymous sentiments
**Presence**	
Being in the moment	Oncologist makes direct comments that indicate they are available and fully in the moment with the patient or family; comments that indicate the oncologist is not rushed and is purposefully giving their time to the patient or family
Silence	Code for any uninterrupted pause, in response to an emotion, that is 5 s or longer in length; pause with intention to create space for processing
**Partnering**	
Nonabandonment	Statements that indicate oncologist will be there to share the entire life or clinical experience with the patient or parent; in it for the long run
Team mentality	Any time statements are used that align or join the oncologist, patient, and family in collaborative goals and decision-making; includes “we” statements, indicating oncologist and family are a team or unit
Accommodating	Discussion of logistics that anticipate needs or accommodate life events for the patient or family
**Inclusivity**	
Open door	Oncologist uses open-ended language that prompts discussion of patient’s or family’s hopes, wishes, opinions, or goals of care; may be in relation to treatment options, location of care, or end-of-life preferences (do not code actual goals of care content)
Affirming	Statements that validate patient or parent as important in decision-making and integral to the process
Connecting symptoms	Oncologist links patient’s symptoms or pain with scan results or disease progression to provide clarity or understandable medical information
Using analogy	Oncologist uses an analogy or a prop to provide clarity or understandable medical information
Showing images	Oncologist shows the patient or family the imaging findings (or will show in the near future) to provide clarity or make medical information more understandable
**Humor**	
Comedy	Oncologist use of comedic relief, attempt at humor, and joking during conversations
Ribbing	The use of playful teasing by the oncologist
Matching maturity level	Oncologist matches the tone, language, and maturity level of a patient to connect with them
**Honesty**	
Warning shot	Oncologist opens with a statement that gives patient or family a moment to emotionally prepare for hearing bad news
Transparency	Oncologist uses statements that attempt to transmit or highlight realistic prognostic assessment related to delivering good or bad prognosis; a linguistic choice that captures oncologist attempt to bond through transparency; includes language about being honest or “I worry” statements
Giving opinion	Oncologist uses statements of ownership, including “I think,” “I feel,” “I recommend,” or “I believe,” or synonyms, while discussing illness course, treatment plan, goals of care; statements that show ownership or personalize opinion while building alliance or partnership with patient or family; includes any time the oncologist uses the phrase “If it were me or my child…”
Summarizing	The oncologist uses a summary statement to reiterate results, treatment, or findings of scans

### Data Analysis

Coding processes were conducted within MAXQDA, a mixed-methods data analysis software system.^25^ Three analysts (E.C.K., S.R., and C.W.) pilot tested the codebook across a series of medical dialogue recordings to identify areas of variance. All analysts met to reconcile variances and achieve consensus, modifying the codebook as needed to improve dependability, confirmability, and credibility of independent codes.^26^ Following codebook finalization, independent coding was performed by 2 analysts (S.R. and C.W.), with weekly meetings to review any coding variances and third-party (E.C.K. and J.N.B.) adjudication to reach consensus. Consistency in code segmentation also was reviewed to ensure a standardized approach (E.C.K., S.R., and C.W.). Code frequency, temporal duration, and distribution across discussion subtype were calculated and reported as descriptive statistics. Codes with greater frequency and impactful content were examined more closely, with further inductive exploration, categorization, and synthesis of identified quotes to generate themes (E.C.K., M.E.L., J.N.B., and J.W.M.). Identified themes were then incorporated into a summary figure, with input from all authors and iterative revision, to ensure that themes were appropriately integrated.

## Results

In total, 33 patient-parent dyads were enrolled and followed longitudinally. From this cohort, 17 patient-parent dyads, representing all 6 participating oncologists, experienced advancing disease during the study period. Most patients were female (11 [64.7%]) and White (15 [88.2%]) individuals; additional participant demographic variables are presented in Table 3. In total, 141 disease reevaluation conversations were audio recorded, comprising approximately 2400 minutes of recorded dialogue. A median of 7 medical discussions per patient (range, 1-19) were recorded. Most patients (14 [82.4%]) died during the study period; 3 remained alive at 24 months. No participants formally dropped out of the study, although 1 dyad received care at another institution prior to death. Data on patient-parent dyads who declined enrollment in the larger study have been published previously.^20^ In brief, 7 of 41 approached dyads (17%) did not enroll owing to hesitation or refusal by either the patient (n = 4) or parent (n = 4). Although these numbers are small, refusal rates did not appear to disproportionately exclude dyads based on race and ethnicity. Only 1 of the 7 dyads self-identified as Black (approximately 14%) and 1 as Hispanic (approximately 14%), which are roughly equivalent to the percentages of Black patients and Hispanic patients treated at the institution.

**Table 3.** Participating Patient, Parent, and Oncologist Characteristics

Variable	No. (%)
Patient	
No.	17
Gender	
Female	11 (64.7)
Male	6 (35.3)
Race	
White	15 (88.2)
Black	1 (5.9)
Multiracial	1 (5.9)
Ethnicity	
Hispanic	0
Non-Hispanic	17 (100)
Age at diagnosis, y	
0-2	2 (11.8)
3-11	6 (35.3)
12-18	7 (41.2)
≥19	2 (11.8)
Parent	
No.	17
Gender/role	
Female/mother	14 (82.4)
Male/father	3 (17.6)
Pediatric oncologist	
No.	6
Gender	
Female	3 (50)
Male	3 (50)
Race	
White	6 (100)
Black	0
Ethnicity	
Hispanic	0
Non-Hispanic	6 (100)
Years in clinical practice	
1-4	2 (33)
5-9	2 (33)
10-19	0
≥20	2 (33)

Across the advancing illness course, we identified 28 unique concepts as potential facilitators associated with therapeutic alliance, with further synthesis generating 7 core themes: human connection, empathy, presence, partnering, inclusivity, humor, and honesty (Table 4). We present these themes as a framework for potential facilitators associated with therapeutic alliance in pediatric cancer (Figure).

**Table 4.** Therapeutic Alliance Codes and Representative Quotes

Code	Example
**Human connection**	
Remembering	And you went and you did the hunt.… How was the hunt, by the way? [Patient: It was awesome.] I’m very glad that you got to do that and had a good time. How was your trip?
Sharing	We almost went there for my kids’ partial spring break because they would just love all the cheesiness. [Parent: So how’s your new little one? Your little nugget.] She’s a mess.… It’s all good though.… My husband was an only child, and so I’m amazed that I got this far. But I had set my sights on that one. I might get a cat. [Patient: Get one.] My daughter wants us to get one. [Patient: Get one.]
Friendly conversation	Wow. [Patient: Hey!] Hey, how are you? [Patient: Good.] Man, you’re getting big. That is cool. Who’s that? Who’s on your shirt? … Look at all this. Wow. I’m going to have to make a request for her to draw something for me. [Parent: She’s going to do the teen art show.… That’s what she’s working on right now.] I’m looking forward to that.
Affection	Bye. [Parent: Bye, Dr X. Patient: I love you, Dr X.] Can I look at your beautiful eyes? Can you just come over here, just for me, just for a second? Alright, bye, sweetie, bye.… Love you. [Parent: Love you.] We’ll see you soon, in 6 weeks.
**Empathy**	
Standing in another’s shoes	I’m glad he’s in a good mood. It made me sad to see him so grouchy. You know, I know it’s hard. There’s no easy way. And our whole clinic felt this way. Everyone was very bummed, just sad when we saw these results.
Naming	[Patient: My heart’s beating really fast, and I don’t know why, and it’s like beating …] You’re probably very anxious. [Patient: It’s beating so fast that it’s, like, making me feel bad, and my stomach is like …] You’re anxious. You just look a little overwhelmed. You looked awfully worried when I walked in the door. Is that what was, is that what you were thinking we’d be talking about today? [Patient: Yeah.] You did. You had a feeling.
Understanding	[Parent: She’s scared now.] Of course. That last therapy was starting to make you feel miserable. [Parent: Yeah.] And that is not worth it because then you’re not getting to do the fun things that your dad was saying that you want to do.
Respecting	So your scans look okay. Let me tell you what I found, okay? I don’t want you to start freaking out. [Parent: Have you dealt with any cases like this before…?] Absolutely. We do that all the time. If you’re thinking of those things and they’re keeping you up at night, you call or email me. [Parent: Okay.] Because we can.… You don’t have to wait, right? I can maybe talk with you. [Parent: There’s still a chance though, right?] There’s always a chance.
Supporting	Sounds like many, many, many providers, all with different or all with ideas … [Parent: They didn’t work well together.] … Okay, so we need to fix that. I don’t even know if this is doing anything or not, but you wanted to do it, and I support 100% if we’re going to do that. And you have all the people that are in this room, and a gazillion that are not in this room, [who] support you 100%.
Exploring	So, we talked a little bit last time about our thoughts with that. I want to kind of pause before we get to, to much else, and ask you what your thoughts and feelings are right now. Are you anxious or nervous or you just can’t sleep or what is it? So tell me, what’s been happening that you are concerned about?
Saying sorry	You know, we’re.… You guys know we’re sorry that we’re finding ourselves in this position. That’s not fair that it happens to you. We’re so sorry. And I know it’s frustrating as well. So I, I, I apologize, you know. So all I can say is I’m sorry. There’s no way around that.
**Presence**	
Being in the moment	I don’t know. Let me go find them. I will have … I will make time. I’m here all next week and Monday through Thursday, and if I need to run down to see you on a Tuesday, that’s fine with me. I don’t … I don’t ever have a problem with that. Of course, that’s going to happen. You just tell us when. That’s what we’re here for.
Silence	NA
**Partnering**	
Nonabandonment	We’re going to be there all the way, regardless of what you decide to do. If she goes and has treatment there, it doesn’t mean that she can’t come back here. She’s our patient. We’re not allowing, we’re not allowing her to leave us unless, you know, unless she’s getting something and promises to come back. But there’s no way to ever know until we go for it, right? We go as hard as we can and nobody’s stopping that. So I don’t want you to feel at all like anybody is saying, “Oh, we’re done and we’re not going to keep trying.” No. We’re going to keep trying for sure. Without fail, okay?
Team mentality	I want you to live, and I want you to graduate. And that’s why we will do whatever you want us to do as much as you want us to do to try to make that happen. It would be nice if we got the next set of scans and things were the same or slightly better. You know, we’ll take whatever we can get. What I would be happy to do today is to say that we probably shouldn’t make a decision today. That I think we should take a little time.
Accommodating	[Patient: Do you know when we can leave?] Today. [Patient: ‘Cause I was invited to go to something.] Go. What are you doing here? I can always give you a call, right, with the results.… It’s not like you have to be here for me to tell you it looks like this, so go. Wednesday would be the best, right, because then your last day would be Christmas eve. You could have Christmas without having to go to the hospital, and then you guys could hit the air. I assume you want to get your chemo done before Thanksgiving? So we could start it tomorrow night in the Medicine room and try to move it up so that by Tuesday we’re done.
**Inclusivity**	
Open door	There’s not a wrong answer for you at this point in terms of whether you want to do something that you feel is more aggressive or whether you want to do something that is taking a step back and focusing a little bit more on, you know, allowing you to do something that gives you some treatment without significantly impacting your quality of life and your ability to do things that you want to do. There are some other things that we can try.… You know, they are available. They are at your disposal, and I’m willing to pursue those things as long as they are things that you want to do and if we feel that it gives us any opportunity to have some benefit.
Affirming	We should use the morphine. If you think that’s what’s best for her. So, it’s a matter of what your goals are for him. In terms of treatment and treatment effects in what you … how you want him to spend the time that he has here with us. I wanted to present you the 3, all 3 options and get a sense of what you felt like, what you felt like was important for you right now and how you perceive all 3 of them.
Connecting symptoms	The biggest one is this one, which is why she complains here. But this one, this one, this one, this one, and this one, and you said that sometimes she complains of pain here, that’s exactly where it is. I think it likely is from one of the, from that area over here, cause I think, just based on the location that you’re reporting, it looks fairly consistent with that region. There’s also some uptake that’s up in the skull base. Umm, and you know, it’s possible that could be contributing to some of the headaches that you’re having because you’re saying that they’re starting at the back of your head.
Using analogy	It’s kind of smushing on. So if this is your—may I borrow your tape? Okay perfect, great. So if this—here look. This is your back bone and then in the middle you have a spinal cord. Okay, so this is your vertebral bodies bones kind of surround your spinal cord, right? We need them to because they protect it. So the tumor that’s growing—we use it with this green tape—it’s kind of growing in this area, and some of it is growing outside of the bone, from the bone and then a little bit is kind of pushing, it’s like kissing the spinal cord. That thing hasn’t really changed in size either, it’s just stayed this big. It’s like the sun … just like this circular thing right there. So that hasn’t changed. So there’s a switch, just to put it simply. When tumor cells turn on that switch, T cells don’t recognize these tumor cells. What all of these drugs do is they basically turn that switch off so that T cells can recognize tumor cells and kill them.
Showing images	So let me pull this up, ‘cause it—a picture—I think makes it much easier to see. So on what we’re seeing is, you can see some changes in the areas that we radiated. For one, these 2 are pretty much almost gone, if not completely gone.… So those 2 lesions on the sides had a nice response. So they’re just, they’re 2, and we can, we can pull up the images if you’d like to see what we’re talking about. There’s just these 2. They’re in the back of the upper right lung.
**Humor**	
Comedy (making jokes)	You know the ankle you just treat with RICE. You know that acronym, right? Rest, ice, compression, elevation.… No, not like eating rice. You’re like, “white or brown?” [Parent: Yeah, we eat lots of rice.] I can already hear you now, like sending an email later: we’ve been eating lots of rice but his ankle still hurts! [Parent: Either that or get somebody from the Medellin cartel to bring it down up.] I don’t know which cartels are active these days.… Honestly, you know, ever since that guy, what’s his name? The guy that finally got caught.… Yeah, El Chapo, I mean, I mean if El Chapo’s not gonna do it, I don’t know who’s gonna get the drug to you. You could tweet it to us. If you could fit it into 140 characters.
Ribbing (gentle mocking)	I’m sorry, every time I look at those pants, it looks like you have [a] beer cozy on your leg. But it sounds like it’s not bugging you. Unless you’re not fessing up. Cough it up, dude.
Matching maturity level	Right, are you still mad at me? Okay, that’s okay. Everybody gets mad at me. I don’t mind. [Patient: It’s okay.] It’s okay. Alright, you forgive me? [Patient: No, no, I was just saying it’s okay.] Oh, okay, so you don’t forgive me. You just saying it’s okay. Gotcha, alright. I know. You’re like, he never probably thought we would be like, come on [patient], we need you to poop! You did it big boy. Thank you. You’re sooo good … Yayyyyy, woohooo! Up here, you wanna come play, come play.
**Honesty**	
Warning shot	Alright, well unfortunately there’s no good way to tell you this. So, I do not have good news. So scans, we got, we got a spot we gotta talk about, okay?
Transparency	[Patient’s name], the bottom line from the scans that we just got is that we’re seeing evidence of progression and that we’re going to come off the study, okay? We’ll do everything all over again and then, you know, periodically for a couple years to make sure. because it still could come back.… As best we know, if 1 cell escapes all this, it eventually comes back, and that’s the scary part. I worry that, because of all these people here, you’re worried more about them than [about] you. Are you?
Giving opinion	In the absence of symptoms, I don’t think that we need to do anything, so I would feel very comfortable just waiting 2 months and repeating the PET. And you know what I’d say to that, we’re not going to do that. [Patient: That’s crazy.] ‘Cause that’s crazy. That’s a terrible idea, okay? So that one is off the table. For me, I think that one makes sense for you because there’s some biological reasons that I think could be effective for [disease type] and because it’s at least a regimen that I think you know you’ve tolerated before.… So I feel like it’s something that could be doable for you.
Summarizing	The long and short of it is, there’s nothing that looks like cancer. So that’s a long way of saying, yes, I’ll consider it, you know, with the caveat of that we gotta get to a state where we feel it’s even worth your time. And so to me that means that the tumor cells are dying, right? They’re not as active, they’re not replicating as much and growing, and so that’s a good thing.

Abbreviations: NA, not available; PET, positron emission tomography.

**Figure.**
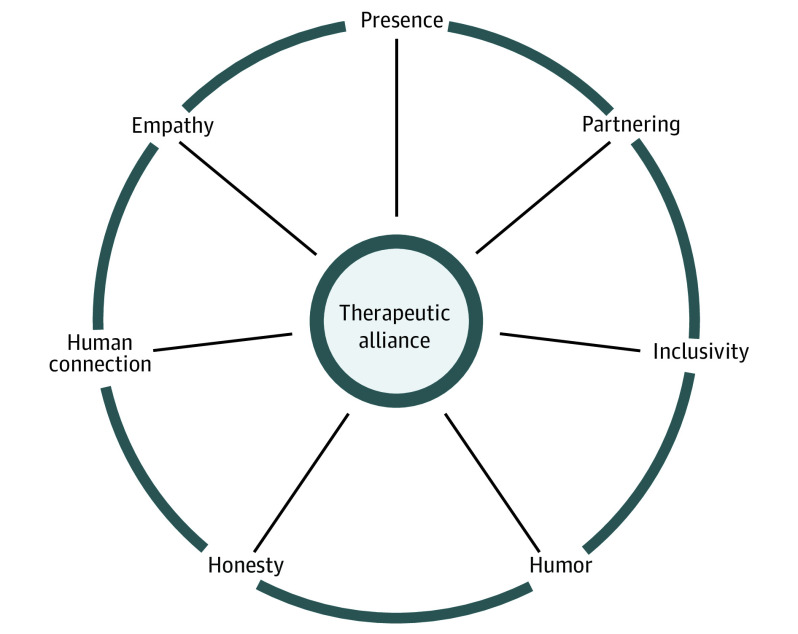
Oncologist Approaches to Building Therapeutic Alliance in Pediatric Cancer Oncologists develop and deepen therapeutic alliance through 7 thematic approaches: human connection, empathy, presence, partnering, inclusivity, humor, and honesty. We present a framework for conceptualizing the interconnectivity and interdependency of these core facilitators associated with therapeutic alliance in pediatric cancer. The framework aesthetic intentionally resembles a wheel, evoking the idea that therapeutic alliance helps to move communication forward more productively, enabling clinicians, patients, and families to travel collaboratively across the illness course.

### Human Connection

Oncologists promoted connection on a human level with the patient and family through 4 approaches. In the *remembering* approach, oncologists recalled information, unprompted, related to the patient’s or family’s personal life. Some recollections were lighthearted or fun (“Where is my birthday twin?”); other times, oncologists recalled prior feelings central to the patient’s values related to decision-making (“I know that in the past, you’ve also said it’s very hard to be away from family.”). Through *sharing*, oncologists also contributed personal information about themselves or their lives as an approach to find common ground with the patient or family. Shared information ranged from minor details about their personality or likes or dislikes (eg, “I’m not a reptile person.”) to a window into their personal life or emotions (eg, “I don’t know about you guys, but the uncertainty drives me crazy.”). Oncologists used *friendly conversation* or small talk unrelated to the illness or treatment plan to connect with patients and families. Finally, *affection* or expressions of fondness were also used to help generate connection on a human level: “It’s okay if you don’t want to, sweetheart.”

### Empathy

We identified 7 facilitators of empathy, including 2 de novo concepts and 5 concepts originating from naming, understanding, respecting, supporting, and exploring, each of which was refined during the inductive coding process. In the first de novo concept, *standing in another’s shoes*, oncologists used empathetic statements to convey a willingness to imagine the patient’s or family’s struggle: “You guys are so wonderful. And I can only imagine how hard this is too because you’ve lost a lot of friends.” In the second de novo concept, *saying sorry*, oncologists used “I’m sorry” statements as a mechanism of conveying empathy: “I’m so sorry . . . that’s not what we wanted to talk to you about today.”

Oncologists also used *naming* to identify emotions that presented during discussion, such as fear, sadness, or anxiety. By labeling the reaction or feeling (eg, “You just got the most nervous look on your face” or “The tears are worries, I got it; it could be [disease]”), oncologists showed an ability to recognize the feelings of the patient or family. Oncologists also expressed *understanding* by acknowledging or appreciating the patient’s and family’s difficult situation and validating their emotions. At times, oncologists (or clinicians) repeated statements as a form of affirmation: [Patient: “That’s a lot of information.”] “It is a lot of information.” Oncologists showed *respect* through provision of encouragement, praise, or reassurance: “You did everything right.” Respect often overlapped with the concept of *supporting*, in which oncologists expressed concern alongside a willingness to help: “As long as you want to try something, we’re willing to try something.” Oncologists also cultivated empathy through the concept of *exploring*, in which they offered patients or family members an opportunity to talk about whatever they were feeling or processing: “What do you want to, what’s running through your head?”

### Presence

We found 2 concepts that promoted a sense of meaningful presence as part of nurturing therapeutic alliance: *being in the moment* and *silence*. Oncologists verbalized with intention their willingness to stay in the moment, whether by giving time (eg, “We need to take the time. I think you guys deserve the time”) or by silencing other distractions (eg, “I turned my ringer off”). Silence was also used with intention to create space for processing and emotional connection.

### Partnering

We discerned 3 concepts that fostered a sense of partnership among oncologists, patients, and their families. In the concept of *nonabandonment*, oncologists expressed a commitment to stay, no matter how difficult things became: “You know that you will never shake us, like good luck trying.” This concept of being “in it for the long run” also manifested through running imagery: “We love you, and we’re going, we’re all going to do this together, okay. This is a marathon and we’re all going to just keep running.” In the concept of *team mentality*, oncologists used “we” language to demonstrate a sense of teamwork and collaboration with respect to values, goals, or decision-making*:* “This is not a conversation that says that we don’t keep fighting because we always keep fighting.” Finally, in the concept of *accommodation*, oncologists partnered with patients and families by anticipating and meeting their needs and preferences. For example, the oncologists offered to reschedule visits or therapies to optimize quality of life: “I think we can do [the treatment] in a way that’s not going to interfere with the trips that you have planned.”

### Inclusivity

We found 5 concepts that centered or included the patient or family with respect to transfer of knowledge or navigation of decision-making. In the *open door* concept, oncologists used open-ended statements to lay groundwork for sensitive conversations and to prompt patients or families to voice their hopes, wishes, opinions, or goals: “If that’s part of our goal . . . maybe we want to do that without getting into really intense things that are going to get you stuck in the hospital and things like that. You know, to me that seems like it would be a very, that would be a very reasonable goal, a good goal to have.” Oncologists also reinforced inclusivity by *affirming* the voice of the patient or parent as integral to decision-making: “Buddy, every decision that we make is the right decision for you because they’re your decisions, you know. We’re not, you know, whatever you decide is not ever going to be the wrong choice.”

Practically, oncologists used several concrete strategies to center the patient or family in medical dialogue. By *connecting symptoms*, they linked imaging findings with how a patient felt, translating abstract data into meaningful patient-centered information: “The biggest issues are in your bones. I think that’s why you feel bad.” By *using analogies*, oncologists simplified medical information into lay language for patients and families: “Your bone can look a little bit more like Swiss cheese, to use an analogy, and then the trouble with Swiss cheese is it has holes, and so if pushing on it having a lot of stress you can sometimes get a little bit of collapse.” Oncologists further included patients and families by *showing imaging* during discussions: “All this area over here is in her right shoulder, even though we radiated it, it’s much, much worse. All this super black stuff, that is disease.”

### Humor

We identified 3 concepts related to humor as a key aspect of therapeutic alliance. In the concept of *comedy*, oncologists made jokes, often at their own expense, sharing in laughter with patients and families. Even serious conversations were sometimes punctuated by moments of levity or silliness via verbal or physical humor or sometimes through song. In the concept of *ribbing*, oncologists connected with patients or families through playful teasing: “She doesn’t have time for this. She’s got things to do. [Parent: Bother me for 1 of them.] Mm-hmm, bother, bother [patient]. That’s top, top of the list every day.” In the concept of *matching maturity level*, oncologists connected with patients by aligning their tone or language choices with the patient’s developmental stage: “I know that’s what you’re thinking, isn’t it? Yeah, like, loser, why haven’t we talked about this before?”

### Honesty

We identified 4 concepts around honesty as a strategy to engender trust. In the *warning shot* concept, oncologists prefaced bad news with a forewarning to allow a patient or family to emotionally prepare: “So I’m afraid I don’t have very good news for you. So let me start from the beginning, okay.” In the concept of *transparency*, oncologists emphasized a desire to be honest or frank, particularly when discussing difficult information: “But it doesn’t mean that we don’t have plans. Okay, I just want to be honest.” Phrases such as “I worry” also enabled oncologists to share their thoughts candidly. Oncologists further fostered alliance via the *giving opinions* concept, using statements of ownership (eg, “I think,” “I feel,” “I recommend,” and “I believe”) when discussing illness progression, treatment planning, or goals of care: “I think we need to continue this therapy. Whether it’s really doing a lot or not, I don’t know, but I feel very uncomfortable stopping.” In the concept of *summarizing*, oncologists synthesized challenging information, thereby strengthening its trustworthiness: “What I would say, then, to kind of put the whole picture together, is that things are stable.”

## Discussion

Therapeutic alliance is one core component of patient- and family-centered communication, particularly in the context of advancing illness. Through analysis of key conversations among pediatric oncologists, patients with advancing cancer, and their families, we identified 7 themes informing a framework for potential facilitators associated with therapeutic alliance: human connection, empathy, presence, partnering, inclusivity, humor, and honesty (Figure). This framework has possible utility in clinical practice and future research. When striving to create, sustain, or repair therapeutic alliance, particularly during stressful time points across an advancing illness course, clinicians might draw from this framework to emphasize certain facilitators with intention. Recognizing that personalities and communication styles naturally vary, clinicians may review the framework and choose to highlight behaviors that feel most genuine to them. We studied conversations recorded at times of crisis for patients and families, and even in these painful spaces, we found moments wherein clinicians created meaning (even laughter) through connection and caring. We hope that the facilitators underpinning this framework may help guide clinicians in forming meaningful therapeutic bonds, particularly when sharing difficult information, as a way to affirm our shared humanity and communicate to patients and families that their feelings matter.

This study also highlights the need for future investigation of several important, unexplored questions. We observed interactions among oncologists, patients, and their families across evolving illness, and we identified potential facilitators associated with therapeutic alliance. However, the identified concepts reflected our perspectives as clinicians and observers. We did not ask parents and children what they personally valued with respect to therapeutic alliance, and we do not know whether these interactions actually supported the human bond between clinicians and children or their families. We also do not know how cultural background or personal lived experiences may influence oncologists’ comfort levels with using certain facilitators of relationship building. For example, although the concept of “sharing personal information” emerged as a frequent approach, this method may not be appropriate or valued by everyone. Similarly, the act of “giving opinions” may come across as paternalistic by some stakeholders. The constructs that we identified, informing development of this initial theoretical framework, require validation to determine whether they align with what patients and families believe to be most important. Future investigation should consider participatory research methods to engage patients and families in assessing the trustworthiness of constructs and prioritizing communication approaches that they perceive to be most meaningful.

Although preliminary, this framework outlines key components associated with creating therapeutic alliance, filling a gap in the literature. Few existing conceptual frameworks characterize the variables intrinsic to development and evolution of therapeutic alliance in pediatric cancer care. One model derived from the experience of oncologists proposes a cyclical reinforcing association between effective communication and therapeutic alliance across the illness trajectory^15^; however, that model does not propose constructs for building alliance. Several models for therapeutic alliance in psychology and psychotherapy exist, yet their authors acknowledge ongoing challenges with application of the models toward measurement and characterization of therapeutic alliance in medicine.^27,28^

Several quantitative tools have been validated to measure therapeutic alliance between patients and oncologists. For example, The Human Connection scale is a psychometrically validated tool with Likert scale items that measures the extent of patient-reported mutual understanding, caring, and trust with their physicians, which has shown reliability in adult cancer populations.^5^ Yet given the nuance and complexity of therapeutic alliance, a mixed-methods approach that incorporates qualitative analysis has potential utility. Recognizing the potential role that therapeutic alliance plays in influencing patient care, caregiver well-being, and bereavement outcomes, studying therapeutic alliance as a potential modifiable factor is important. This theoretical framework offers potential infrastructure to further our understanding of therapeutic alliance in future cancer research.

### Limitations

Study limitations include single-site design and potential sample bias for patient-parent dyads inclined to value or promote therapeutic alliance; all eligible oncologists participated, mitigating selection bias for physicians. Racial and ethnic diversity was limited and requires prioritization in future work. Rarely, discussions were not recorded due to logistical issues or at the request of the participating patient or parent; missing data might influence concept generation. However, given the saturation of themes across thousands of recorded minutes, several missing time points are less likely to influence the synthesis of findings.

## Conclusions

Therapeutic alliance is an important aspect of the illness and care experience for children with cancer and their families. Drawing from longitudinal medical dialogue across an advancing illness course, we identified 7 core themes associated with therapeutic alliance: human connection, empathy, presence, partnering, inclusivity, humor, and honesty. These findings offer a framework to support clinician education and future opportunities to study key stakeholder perspectives on approaches to optimize therapeutic alliance among oncologists, patients, and their families.
